# Initiation of health-behaviour change among employees participating in a web-based health risk assessment with tailored feedback

**DOI:** 10.1186/1745-6673-6-5

**Published:** 2011-03-09

**Authors:** Ersen B Colkesen, Maurice AJ Niessen, Niels Peek, Sandra Vosbergen, Roderik A Kraaijenhagen, Coenraad K van Kalken, Jan GP Tijssen, Ron JG Peters

**Affiliations:** 1 Department of Cardiology, Academic Medical Center - University of Amsterdam, P.O. Box 22660, 1100 DD, Amsterdam, The Netherlands; 2 NDDO Institute for Prevention and Early Diagnostics (NIPED), Amsteldijk 194, 1079 LK Amsterdam, The Netherlands; 3 Department of Medical Informatics, Academic Medical Center - University of Amsterdam, P.O. Box 22660, 1100 DD, Amsterdam, The Netherlands

## Abstract

**Background:**

Primary prevention programs at the worksite can improve employee health and reduce the burden of cardiovascular disease. Programs that include a web-based health risk assessment (HRA) with tailored feedback hold the advantage of simultaneously increasing awareness of risk and enhancing initiation of health-behaviour change. In this study we evaluated initial health-behaviour change among employees who voluntarily participated in such a HRA program.

**Methods:**

We conducted a questionnaire survey among 2289 employees who voluntarily participated in a HRA program at seven Dutch worksites between 2007 and 2009. The HRA included a web-based questionnaire, biometric measurements, laboratory evaluation, and tailored feedback. The survey questionnaire assessed initial self-reported health-behaviour change and satisfaction with the web-based HRA, and was e-mailed four weeks after employees completed the HRA.

**Results:**

Response was received from 638 (28%) employees. Of all, 86% rated the program as positive, 74% recommended it to others, and 58% reported to have initiated overall health-behaviour change. Compared with employees at low CVD risk, those at high risk more often reported to have increased physical activity (OR 3.36, 95% CI 1.52-7.45). Obese employees more frequently reported to have increased physical activity (OR 3.35, 95% CI 1.72-6.54) and improved diet (OR 3.38, 95% CI 1.50-7.60). Being satisfied with the HRA program in general was associated with more frequent self-reported initiation of overall health-behaviour change (OR 2.77, 95% CI 1.73-4.44), increased physical activity (OR 1.89, 95% CI 1.06-3.39), and improved diet (OR 2.89, 95% CI 1.61-5.17).

**Conclusions:**

More than half of the employees who voluntarily participated in a web-based HRA with tailored feedback, reported to have initiated health-behaviour change. Self-reported initiation of health-behaviour change was more frequent among those at high CVD risk and BMI levels. In general employees reported to be satisfied with the HRA, which was also positively associated with initiation of health-behaviour change. These findings indicate that among voluntary participating employees a web-based HRA with tailored feedback may motivate those in greatest need of health-behaviour change and may be a valuable component of workplace health promotion programs.

## Introduction

Cardiovascular diseases (CVD) are the leading cause of disability and death[1]. Much of the CVD burden could be eliminated by addressing preventable risk factors, including high blood pressure, hypercholesterolemia, hyperglycaemia, smoking, physical inactivity, high fat intake, and low fruit and vegetable intake[2,3]. The health risk assessment (HRA) is one of the most widely used strategies to stimulate changes in these factors[4-6]. The worksite has been proposed as a suitable platform for wide dissemination of prevention programs that utilize HRA, with the advantage of cost savings, the creation of a health-conscious environment and easier follow-up of high-risk individuals[7,8].

The traditional HRA screened for risk factors to produce feedback that predominantly contained information on the assessed risk[9]. However, reviews of the literature did not always support effectiveness of the traditional HRA[9,10]. It was suggested that feedback merely containing risk information would be insufficient to initiate health-behaviour change[11]. It was acknowledged that improvements in affecting health-behaviour change could be achieved by web-based delivery of the HRA, with incorporation of tailored health recommendations[11-14]. These HRAs hold the advantage of simultaneously increasing awareness of risk and enhancing initiation of health-behaviour change[11,15].

Despite this potential little has been documented regarding health-behaviour change after implementation of a web-based HRA with tailored feedback at the workplace. In the present study we evaluated initial health-behaviour change among employees who voluntarily participated in a web-based HRA including tailored feedback, offered to them by their employer as part of a worksite health management program. The HRA was designed to collect data that are necessary to screen for the risk of a number of preventable diseases, including CVD, and provide tailored feedback to educate, motivate and empower participants to engage in a better lifestyle and reduce CVD risk. The primary aim of this study was to assess self-reported initiation of health-behaviour change and associations with satisfaction with the HRA and baseline health status.

## Methods

### Population and study procedure

We conducted a questionnaire survey among employees who completed a web-based HRA with tailored feedback. This HRA was applied as part of a worksite health management program at seven Dutch companies with mainly white-collar workers between 2007 and 2009. During this period 6790 employees were invited to complete the HRA. E-mail invitations were sent by the human resources department, with a single reminder after two weeks. The invitation e-mail included a description of the HRA and informed employees that participation was voluntary, at no cost, that all personal data would be treated confidentially, and that no results would be shared with their employer or any other party. Employees who completed the HRA, were sent an electronic satisfaction and health-behaviour change questionnaire, four weeks after they had received their tailored feedback. The questionnaire measured overall satisfaction with the HRA and initiation of health-behaviour change. It was sent to the employees using an e-mail survey program, with a single reminder after one week, and took about 10 minutes to complete.

### The web-based HRA with tailored feedback

The HRA consisted of four components: 1) a web-based electronic health questionnaire, 2) biometric measurements, 3) laboratory evaluation, and 4) tailored health recommendations, based on the results of the first three components. The electronic health questionnaire includes approximately 100 questions covering socio-demographics, personal health history, family risk, and the behavioural domain. All questions are derived from validated questionnaires and health-behaviour constructs from the transtheoretical model,[16] protection motivation theory,[17] and social cognitive theory[18]. Biometric measurements (length, weight, waist circumference, blood pressure) are conducted at the worksite by trained and certified staff, usually staff of the occupational health services provider of the employer. Measurements are directly entered in the central HRA database. At the same visit blood samples are collected for laboratory testing of total cholesterol, HDL, LDL, triglycerides, glucose and HbA1C. Collected samples are shipped to a certified laboratory where analyses are completed and results are electronically transferred to the central HRA database. For system security and data protection reasons personal identification data and risk assessment data are stored on separate servers. An electronic firewall is placed between the servers and the Internet. Only users certified by ID and password are able to access the servers. By computer-based combination of the assessed risk with health-behaviour constructs, tailored health recommendations are generated. These are presented to the participant integrated within a web-based health action plan. Each health plan comprises: 1) explanation of the assessed risk for each of the targeted preventable conditions, using a three-colour system (green: normal risk profile; orange: moderately elevated risk profile; red: seriously elevated risk profile), 2) explanation of the threats associated with elevated risk and potential gains of taking preventive action, and 3) opportunities for taking preventive action based on the participant's stated motivation for health-behaviour change (physical activity, smoking cessation, alcohol intake, dietary habits), self-efficacy, and preferences with respect to interventions (e.g. guided vs. non-guided interventions). Where possible, recommendations are based on prevailing practice guidelines. For example, cardiovascular risk factor cut-off values are derived from the European and Dutch guidelines for cardiovascular risk management[19,20]. When seriously elevated risks are detected, the health plan includes referral for further medical evaluation and treatment. A 30 minute health counselling session with the program physician is also available upon request for all participants.

### Satisfaction and initiation of health-behaviour change questionnaire

The study questionnaire included seven questions examining satisfaction with the web-based HRA and initiation of health-behaviour change after receiving the tailored health advices. An outline of the items, questions, and scoring scales are shown in the Additional file 1. Satisfaction was measured with two questions, using evaluative statements on the program as a whole: 1) overall mark for the program, measured on a 5-point rating scale, and 2) recommending the program to others, measured on a 5-point agreement scale. Initiation of health-behaviour change was measured with one item that evaluated whether participants overall initiated health-behaviour change after receiving their health advices, followed by questions on which health-behaviour items change was initiated. Answer options were yes, no, and not applicable.

### Analysis

All analyses included descriptive statistics to examine population characteristics, and questionnaire answers for satisfaction and initial health-behaviour change. Non-response bias was checked by comparing differences in baseline values between responders and non-responders to the study questionnaire, using chi-squared tests. To analyze the influence of demographic factors and health characteristics on satisfaction with the HRA, logistic regression analysis was performed, with dichotomized Likert scale responses in positive and negative evaluation as dependent variable and the variables of interest (age category, sex, education level, body mass index as a proxy for physical activity level and caloric intake, smoking status, and Framingham CVD risk score as a proxy for cardiovascular risk factor levels) as covariates. The Framingham score estimates 10-year CVD mortality and morbidity risk by combining age, sex, blood pressure, hypertension treatment status, total cholesterol, HDL-cholesterol, smoking and diabetes status[21]. CVD risk score was categorized in low, intermediate and high risk, defined as 10-year CVD risk of <10%, ≥10% to 20% and ≥20%. The influence of satisfaction with the HRA program and health characteristics on initial health-behaviour change was also examined using logistic regression. All analyses were adjusted for age, sex, and education level. Data were analyzed using SPSS for Windows, version 17.

## Results

Of the 6790 invited employees, 2289 (34%) completed all HRA measurements and received tailored health advices. Approximately 30 days after receiving health advices all 2289 employees were sent the study questionnaire. The response rate was 28% (638/2289). There were no differences between employees who responded to the questionnaire and those who did not in sex, age category, education level, Framingham risk score, body mass index, and smoking status (see Table 1). In Tables 2 and 3 results of the questionnaire are summarized. Of all employees who responded to the questionnaire 86% gave a positive overall rating and 74% recommended the program to others. Overall, 368 (58%) employees reported to have initiated health-behaviour change, 242 (38%) to have improved physical activity, 64 (10%) to have reduced alcohol intake, and 282 (44%) to have improved their diet. Twenty employees reported to have quit smoking, representing 14% (20/145) of all current smokers among the questionnaire responders.

**Table 1:** Baseline characteristics of employees who completed the HRA and responded to the satisfaction and health-behaviour change questionnaire and those who completed the HRA but did not respond the questionnaire.

	questionnaire responders n = 638	questionnaire non-responders n = 1651	*p*
Sex			
Male	387(61%)	1017(62%)	*0.679*
Female	251(39%)	634(38%)	
Age Category			
<30 years	28(4%)	89(5%)	*0.054*
30-39 years	163(26%)	457(28%)	
40-49 years	233(37%)	646(39%)	
>50 years	214(34%)	459(28%)	
Education level			
Low	139(22%)	320(19%)	*0.204*
Midlevel	191(30%)	552(33%)	
High	308(48%)	779(47%)	
Framingham 10 year CVD risk score category			
Low CVD risk (Framingham score < 10%)	455(71%)	1213(73%)	*0.578*
Intermediate CVD risk (Framingham score ≥ 10% - < 20%)	132(21%)	318(19%)	
High CVD risk (Framingham score ≥ 20%)	51(8%)	120(7%)	
Body Mass Index category			
Normal weight: Body Mass Index < 25 kg/m^2^	349(55%)	885(54%)	*0.248*
Overweight: Body Mass Index ≥ 25 - < 30 kg/m^2^	221(35%)	620(38%)	
Obese: Body Mass Index ≥ 30 kg/m^2^	68(11%)	146(9%)	
Current smoking status			
non-smoker	493(77%)	1272(77%)	*0.907*
smoker	145(23%)	379(23%)	

Values are expressed as number (% of total)

**Table 2:** Satisfaction scores of 638 employees who completed the HRA and responded to the satisfaction and health-behaviour change questionnaire.

	Satisfaction ratings	
	
	Positive	Negative
Overall mark	546(86%)	92(14%)
Recommend to others	473(74%)	165(26%)

Values are expressed as number (% of total).

Positive for the satisfaction item "Overall mark" reflects the proportion rating the item as excellent, very good, or good, and negative reflects the proportion rating the item as average or poor.

Positive for the satisfaction item "Recommend to others" reflects the proportion rating the item as certainly yes or probably yes, and negative reflects the proportion rating the item as maybe, probably no, and certainly no.

**Table 3:** Self-reported initiation of health-behaviour-change of 638 employees who completed the HRA and responded to the satisfaction and health-behaviour change questionnaire.

	Initiation of health-behaviour-change after receiving health advices		
	
	Yes	No	na^†^
Initiated overall health-behaviour-change after receiving tailored health advices	368(58%)	243(38%)	27(4%)
			
More physical activity	242(38%)	212(33%)	184(29%)
Quit smoking	20(3%)	125(20%)	493(77%)
Reduced alcohol intake	64(10%)	198(31%)	376(59%)
Improved diet	282(44%)	158(25%)	198(31%)

Values are expressed as number of participants (%).

na^†^: Questionnaire responders who stated that health-behaviour change on item of interest was not applicable.

In Table 4 the influence of demographic factors and health characteristics on self-reported health-behaviour change are summarized. Age category and sex did not influence self-reported health-behaviour change. Compared to those with a low education level, higher educated employees were less likely to reduce alcohol intake (OR 0.50, 95% CI 0.25-0.99). Compared with employees at low CVD risk, those at intermediate CVD risk more often reported to have started to change their health behaviour in general (OR 1.71, 95% CI 1.04-2.80), whereas those at high CVD risk more often reported to have increased physical activity (OR 3.36, 95% CI 1.52-7.45). Independently, overweight (OR 1.63, 95% CI 1.13-2.36) and obese (OR 1.76, 95% CI 1.00- 3.10) employees more frequently reported initiation of overall health-behaviour change, and to have increased their physical activity (OR 1.56, 95% CI 1.03-2.36 for overweight and OR 3.35, 95% CI 1.72-6.54 for obese). Obese employees also more often reported to have improved their diet (OR 3.38, 95% CI 1.50-7.60). No associations between smoking status and self-reported initiation of health-behaviour change were found. An overall positive satisfaction with the HRA was associated with more frequent self-reported initiation of overall health-behaviour change (OR 2.77, 95% CI 1.73-4.44), increased physical activity (OR 1.89, 95% CI 1.06-3.39), and improved diet (OR 2.89, 95% CI 1.61-5.17). Being positive on recommending the program to others was similarly associated with more frequent self-reported initiation of overall health-behaviour change (OR 2.27, 95% CI 1.57-3.29), increased physical activity (OR 1.65, 95% CI 1.06-2.59), and improved diet (OR 3.00, 95% CI 1.89-4.78). Reported satisfaction with the HRA was not related to demographic factors and health characteristics with (data not shown).

**Table 4:** Influences of demographic and health characteristics on self-reported initiation of health-behaviour change.

	Overall health-behaviour change	More physical activity	Quit smoking	Reduced alcohol intake	Improved diet
	
	OR [95% CI]	OR [95% CI]	OR [95% CI]	OR [95% CI]	OR [95% CI]
Sex					
Male‡					
Female	0.88[0.63 - 1.23]	1.20[0.82 - 1.76]	2.00[0.76 - 5.24]	0.89[0.47 - 1.69]	1.25[0.84 - 1.88]
Age					
40-49 years‡					
<30 years	1.05[0.47 - 2.36]	1.44[0.57 - 3.66]	**	1.67[0.39 - 7.07]	2.04[0.72 - 5.81]
30-39 years	0.92[0.61 - 1.39]	1.14[0.71 - 1.85]	1.66[0.53 - 5.25]	1.54[0.70 - 3.36]	1.00[0.61 - 1.63]
>50 years	1.39[0.94 - 2.06]	0.90[0.58 - 1.39]	0.55[0.17 - 1.83]	1.33[0.68 - 2.59]	1.13[0.70 - 1.81]
Education level					
Low‡					
Midlevel	1.08[0.69 - 1.70]	1.07[0.63 - 1.81]	1.37[0.36 - 5.20]	0.64[0.30 - 1.37]	1.10[0.62 - 1.96]
High	0.99[0.65 - 1.49]	1.20[0.74 - 1.94]	1.10[0.31 - 3.93]	0.50[0.25 - 0.99]	0.64[0.38 - 1.07]
Framingham 10 year CVD risk score (%)					
Low CVD risk (Framingham score < 10%)‡					
Intermediate CVD risk (Framingham score ≥ 10% - < 20%)	1.74[1.10 - 2.74]	1.40[0.84 - 2.32]	1.83[0.48 - 7.02]	1.29[0.63 - 2.63]	1.11[0.65 - 1.90]
High CVD risk (Framingham score ≥ 20%)	1.82[0.92 - 3.59]	2.76[1.29 - 5.90]	3.88[0.80 - 18.75]	1.83[0.72 - 4.63]	1.03[0.47 - 2.29]
Body Mass Index category					
Normal weight: Body Mass Index < 25 kg/m2 ‡					
Overweight: Body Mass Index ≥ 25 - < 30 kg/m^2^	1.63[1.13 - 2.36]	1.56[1.03 - 2.36]	0.89[0.29 - 2.68]	1.69[0.91 - 3.14]	1.44[0.93 - 2.23]
Obese: Body Mass Index ≥ 30 kg/m^2^	1.76[1.00 - 3.10]	3.35[1.72 - 6.54]	2.57[0.42 - 15.81]	1.20[0.45 - 3.19]	3.38[1.50 - 7.60]
Current smoking status					
non-smoker‡					
smoker	1.03[0.70 - 1.51]	0.89[0.58 - 1.38]	††	1.36[0.74 - 2.49]	0.93[0.59 - 1.47]
Satisfaction					
Negative overall mark‡					
Positive overall mark	2.77[1.73 - 4.44]	1.89[1.06 - 3.39]	0.70[0.17 - 2.85]	1.56[0.64 - 3.79]	2.89[1.61 - 5.17]
Negative recommend to others‡					
Positive recommend to others	2.27[1.57 - 3.29]	1.65[1.06 - 2.59]	0.53[0.19 - 1.46]	1.42[0.73 - 2.77]	3.00[1.89 - 4.78]

OR: Odds ratio. 95% CI: 95% confidence interval

‡: Reference category

*: OR could not be calculated because none of the responders at age <30 years reported quit smoking.

†: OR for reporting quit smoking between smokers and non-smokers is irrelevant.

ORs for Framingham score, Body Mass Index, and Smoking status were adjusted for age, sex, and education level.

## Discussion

The present study evaluated self-reported initial health-behaviour change among employees who completed a web-based HRA with tailored feedback. More than half of the employees reported to have initiated overall health-behaviour change. Initiation of more physical activity and improved diet was more frequently reported among those at high CVD risk and BMI levels. In general, employees reported to be satisfied with the HRA, and this was also positively associated with initiation of health-behaviour change.

An important finding in the present study is that employees at higher risk of CVD and high BMI levels more frequently reported initiation of health-behaviour change in general, increase in physical activity and improved diet. These findings may imply that the program is capable of stimulating health-behaviour change among those at greatest need. A possible underlying mechanism may be the tailoring of health advices to individual health characteristics, stage of change[16], motivation[17], and self-efficacy[18]. The feedback provided in the program therefore might be less stigmatizing and better aligned with the intentions of the participants, allowing them to change in small steps. These are factors that were previously associated with poor satisfaction ratings of health services among those at higher risk levels[9,12,14,22,23].

In the present study we found no influence of demographic factors and health characteristics on reported satisfaction with the HRA. These findings are not consistent with previous studies that evaluated satisfaction in the context of a health service. Studies usually associated higher age, female gender, and low educational level with higher levels of satisfaction[22,24,25]. However, previous satisfaction studies generally evaluated a service that was based on face-to-face encounters with health professionals. The web-based HRA program we studied is a highly automated health service that includes a face-to face encounter with professionals upon request or when medically necessary. These characteristics may be relevant in designing HRA programs to reach higher satisfaction, and consequently greater health-behaviour change.

The present study has several limitations. First, the response rate to the questionnaire was 28%, which is lower than the mean response rates of 60% to 67% in most satisfaction surveys[26,27]. However, our response rate is comparable with response rates of general e-mail health surveys, which are around 34%[28]. Moreover, we did not find any differences in demographic and health parameters between responders and non-responders to the questionnaire. Therefore we assume that the sample was representative for all participants of the HRA program. Second, participation in the HRA was voluntary, with a participation rate of 34%. Studies that evaluated HRA or health promotion programs reported participation rates from 20% to 76%,[29,30] with the general impression that females, older employees, and mainly the "worried well" are attracted[31]. Although the participation rate in this study is within the expected range, we cannot rule out that among non-participants in the HRA there were employees with less favourable health characteristics. Third, both satisfaction and health-behaviour change were self-reported and therefore may be due to a number of psychosocial artefacts, including social desirability bias and a novelty effect[22,25]. Finally, the high positive satisfaction rating for overall mark may be skewed, because an unbalanced Likert scale with 3 positive scores and 2 negative scores was used. However, a previous study using a comparable scale reported an overall positive rating of 84%, which is similar with our findings[15]. Furthermore, we found that the item "recommend to others", which was assessed on a balanced scale, was also rated positive by the majority of the participants and had similar influence on self-reported initiation of health-behaviour change. Therefore, we assume that the impact of the unbalanced scale was marginal.

## Conclusion

More than half of the employees who voluntarily participated in a web-based HRA with tailored feedback, reported to have initiated health-behaviour change within four weeks after receiving their feedback. Self-reported initiation of health-behaviour change was more frequent among those at high CVD risk and with high BMI levels. In general, employees reported to be satisfied with the HRA, which was also positively associated with initiation of health-behaviour change. These findings indicate that among voluntary participating employees, a web-based HRA program with tailored feedback could motivate those in greatest need of health-behaviour change. A web-based HRA with tailored feedback could therefore be a valuable component of workplace health promotion programs.

## Competing interests

CKvK and RAK are directors and co-owners of NIPED. This institute developed the studied program and currently markets it in the Netherlands. For the present study NIPED provided for a Ph.D. grant for EBC.

MAJN is a full-time employed as researcher by NIPED. NP is part-time employed by NIPED as head of the research department and part-time employed at the Academic Medical Center - University of Amsterdam as assistant professor. All other authors are employed by the Academic Medical Center - University of Amsterdam. They received no additional funding for this study and report no competing interests.

## Authors' contributions

RJGP and JGPT were the principal investigators of the study, developed the concept and design of the study, and contributed to the interpretation of data. EBC carried out the data collection, data analyses, performed the main writing and drafted the manuscript. MAJN carried out statistical analyses under supervision of NP. EBC, MAJN, and SV drafted the manuscript. RAK, CKvK and NP participated in coordination of the study. All authors reviewed a previous version of the manuscript and vouch for the accuracy and completeness of the data and analyses.

## Funding

A Ph.D. grant for EBC and study materials were funded by NIPED.

## Supplementary Material

Additional file 1 Outline of the study questionnaire. Click here for file
