# Supplementary material for: Initiation of health-behaviour change among employees participating in a web-based health risk assessment with tailored feedback
Source: J Occup Med Toxicol. 2011 Mar 9;6:5. doi: 10.1186/1745-6673-6-5 (PMC3065446; doi:10.1186/1745-6673-6-5)
Supplement: Additional file 1 — Outline of the study questionnaire. [file 1745-6673-6-5-S1.PDF]

Additional file 1. Outline of the study questionnaire.

	Items specification	Scoring*
Satisfaction	Overall mark	5-point rating scale
	Recommend to others	5-point agreement scale
Initiation of health-behaviour change	Initiated overall health-behaviour-change after receiving tailored health advices	yes-no-not applicable
	More physical activity	yes-no-not applicable
	Quit smoking	yes-no-not applicable
	Reduced alcohol intake	yes-no-not applicable
	Improved diet	yes-no-not applicable
